# Intelligent Mechanisms of Macrophage Apoptosis Subversion by *Mycobacterium*

**DOI:** 10.3390/pathogens9030218

**Published:** 2020-03-16

**Authors:** Abualgasim Elgaili Abdalla, Hasan Ejaz, Mahjoob Osman Mahjoob, Ayman Ali Mohammed Alameen, Khalid Omer Abdalla Abosalif, Mohammed Yagoub Mohammed Elamir, Mohammed Alsadig Mousa

**Affiliations:** 1 Department of Clinical Laboratory Sciences, College of Applied Medical Sciences, Jouf University, Al Jouf 2014, Saudi Arabia; hetariq@ju.edu.sa (H.E.); aaalameen@ju.edu.sa (A.A.M.A.); koabosalif@ju.edu.sa (K.O.A.A.); myelamir@ju.edu.sa (M.Y.M.E.); 2 Department of Medical Microbiology, Faculty of Medical Laboratory Sciences, Omdurman Islamic University, Omdurman 14415, Sudan; 3 Department of Chemical Pathology, Faculty of Medical Laboratory Sciences, University of Khartoum, Khartoum 11081, Sudan

**Keywords:** Mycobacterium, macrophage, apoptosis, effector, cytokine, microRNA

## Abstract

Macrophages are one of the first innate defense barriers and play an indispensable role in communication between innate and adaptive immune responses, leading to restricted *Mycobacterium tuberculosis* (*Mtb*) infection. The macrophages can undergo programmed cell death (apoptosis), which is a crucial step to limit the intracellular growth of bacilli by liberating them into extracellular milieu in the form of apoptotic bodies. These bodies can be taken up by the macrophages for the further degradation of bacilli or by the dendritic cells, thereby leading to the activation of T lymphocytes. However, *Mtb* has the ability to interplay with complex signaling networks to subvert macrophage apoptosis. Here, we describe the intelligent strategies of *Mtb* inhibition of macrophages apoptosis. This review provides a platform for the future study of unrevealed *Mtb* anti-apoptotic mechanisms and the design of therapeutic interventions.

## 1. Introduction

Tuberculosis (TB) is a chronic infectious disease caused by *Mycobacterium tuberculosis* (*Mtb*), which affects 10 million people globally. It was one of the top 10 infectious diseases in 2017, with an estimated 1.6 million deaths worldwide, which dropped to 1.5 million in 2018 [1]. *Mtb* is considered a sinful intercellular pathogen that is capable of surviving and replicating within the hostile microenvironment of macrophages and other cellular niches [2]. Macrophages are at the frontline of the innate defense, encounter the pathogens, and play a critical role in the containment of bacilli infection through triggering inflammation, digestion of bacilli, and inducing adaptive immune responses [3]. However, *Mtb* can subvert macrophage responses such as apoptosis in order to establish a persistent lifestyle [4]. Thus, understanding the underlying mechanisms behind *Mtb* manipulation of macrophages apoptosis is decisive for treatment interventions and TB vaccines.

Apoptosis is a highly coordinated and regulated process of the programmed cell death form in which dying cell components and *Mtb* are enclosed within cytoplasmic membranes and liberated from the cell called apoptotic bodies [5]. The apoptosis of macrophages is a crucial part of the innate host defense against *Mtb* through restricting the intracellular growth of bacilli. The *Mtb* infected macrophage undergoes apoptosis in order to release the *Mtb* into the extracellular milieu, thereby leading to the activation of dendritic cells and triggering of robust adaptive immune responses [6,7]. Nevertheless, *Mtb* has evolved multiple mechanisms to revoke macrophages apoptosis, which are discussed in the current review.

## 2. Anti-Apoptotic Determinants of Mycobacterium

*Mtb* has a wide variety of effector molecules that actively block macrophage apoptotic pathways. In this context, cell wall-associated glycolipids, such as lipoarabinomannan (LAM) and mannosylated LAM (ManLAM), are important virulence determinants that have been found to modulate macrophages apoptosis [8,9]. ManLAM can thwart *Mtb* mediating B10R macrophage apoptosis by inhibiting calcium influx and its intracellular signaling. Ablation of calcium-signaling leads to the inhibition of caspase-1 activity, alteration of mitochondrial membrane permeability, as well as induced upregulation of anti-apoptotic, namely, B-cell lymphoma 2 (Bcl2) (Table 1) [8]. LAM can also block human leukemia monocytic cell (THP-1) apoptosis by activating phosphatidylinositol 3-kinase (PI3K), which in turn activates serine/threonine kinase (Akt) that suppresses pro-apoptotic factor Bad (Table 1) [9].

*Mtb* tyrosine phosphatase (PtpA) is a secreted protein that plays a decisive role in the pathogenesis and survival of *Mtb* within macrophages via interplay with multiple host signaling pathways [10]. PtpA knockout *Mtb* strain induces a high level of caspase-3 expression and promotes its activity in THP-1 macrophages when compared with wild *Mtb* strains. PtpA can block caspase-3 activity by dephosphorylation of human glycogen synthase kinase 3 (GSK3) (Table 1) [11]. Similarly, PtpA can attenuate differentiated U937 macrophages apoptosis in response to Bacillus Calmette–Guérin (BCG) strain infections. PtpA can abolish the ubiquitin ligase of the tripartite motif-containing (TRIM) protein activity, which is required for pro-caspase-3 cleavage [12]. Noticeable, another *Mtb* secreted tyrosine phosphatase (MptpB) is a decisive virulence factor that can modulate macrophage responses and enhance *Mtb* intracellular survival. RAW264.7 cells expressing MptpB displayed much less apoptosis than the control cells when infected with BCG strains. Furthermore, interferon-gamma (IFN-γ)-stimulated RAW264.7 cells expressing MptpB show a high viability rate and low levels of cleaved caspase-3 in response to BCG infection when compared RAW264.7 cells-harboring empty vector [13]. Taken together, the anti-apoptotic mechanisms of *Mtb* tyrosine phosphatases are dependent on the inhibition of caspase-3 activation (Table 1).

*Mtb* has 11 types of serine/threonine protein kinases (PknA through PknL) that play a significant role with respect to bacterial adaptation in hostile environments, both in vivo and in vitro [14]. Among them, pknE has anti-apoptotic activity and is indispensable for *Mtb* survival inside the macrophage. PknE deleted *Mtb* strains induce higher levels of THP-1 macrophage apoptosis than the wild type strains and can suppress oxidative stress-inducing cellular apoptosis [15,16]. PknE can modulate the expression of multiple apoptotic molecules and reduce the expression of pro-apoptotic factors, including P53, Bax, and TNF-α, while increasing the expression of anti-apoptotic factor Mcl-1 (Table 1). Furthermore, pknE can promote the phosphorylation of Akt [16], which in turn suppresses the pro-apoptotic factor, i.e., Bad protein [9].

*Mtb* NADH-ubiquinone oxidoreductase subunit G (nuoG), a subunit of type-1 NADH dehydrogenase (NADH-1), was demonstrated to suppressed THP-1 and murine bone marrow-derived macrophages (BMDMs) apoptosis and promoted the pathogenesis of bacilli in a mice infection model [17]. NuoG knockout *Mtb* strains were less virulent and lost their ability to abrogate macrophage apoptosis in comparison with control strains. The ability of nuoG deleted *Mtb* strains to provoke macrophage apoptosis was significantly reversed upon treatment with caspase-3 and caspase-8 inhibitors, or through the ablation of TNF-α signaling. NuoG can suppress NADPH oxidase (NOX2)-inducing of ROS production leading to inhibition of TNF-α secretion (Table 1) [18]. A recent study demonstrates that the deletion of *Mtb* TNF-α-suppressing genes results in increased macrophage apoptosis and promotes a cell-mediated immune response [19]. Therefore, targeting nuoG might improve the immune responses and control the intracellular growth of *Mtb*. 

*Mtb* nucleoside diphosphate kinase (Ndk) acts as a small GTPase inhibitor that can deactivate GTPase Rac1 and, subsequently, inhibit NOX2 assembly and ROS production. Ndk has been demonstrated to block murine macrophage apoptosis by abolishing ROS-mediated caspase-3 cleavage (Table 1) [20].

*Mtb* isocitrate lyase (Icl) is crucial for bacilli viability during latent infection [21]. The recombinant *M. smegmatis-*expressing IcI enhances survival within RAW264.7 murine macrophages and inhibits apoptosis in comparison with *M. smegmatis*-harboring empty vector [22]. Nevertheless, the underlying mechanisms by which IcI subverts macrophage apoptosis are yet be determined.

*Mtb* PE_PGRS family proteins are the most abundant cell wall anchored proteins that are implicated in bacilli adhesion, invasion, and survival within macrophages and dendritic cells [23]. PE_PGRS62, PE_PGRS41, and PE_PGRS18 contribute actively to revoke macrophage apoptosis [24,25,26]. 

The ectopic expression of PE_PGRS62 in *M. smegmatis* enhances its survival within THP-1 macrophages and reduces cell apoptosis. It has been observed that PE_PGRS62 can inhibit the endoplasmic reticulum (ER) stress response (Table 1) by downregulating C/EBP homologous protein (CHOP) and the 78-kDa glucose-regulated protein (GRP78/Bip), which are essential pro-apoptotic factors in response to stress conditions [25]. Likewise, *M. smegmatis*-expressing PE_PGRS41 induces much lower THP-1 macrophage apoptosis than the *M. smegmatis* expressing vector. PE_PGRS41 can decrease the cleavage levels of caspase-9 and caspase-3 (Table 1) [24]. PE_PGRS18 also inhibits THP-1 apoptosis. However the underlying molecular mechanisms are still unknown [26].

*Mtb* secreted proteins encoded by *Rv3654c* and *Rv3655c* genes express within the cytoplasm of *Mtb*-infected macrophages, suggesting that they may have a role in *Mtb* interplays with host cell signaling [27]. These proteins abrogate the extrinsic pathway-mediated U937 cells apoptosis during *Mtb* infection. They interact and cleave the polypyrimidine tract binding Protein-associated Splicing Factor (PSF) result in the deactivation of caspase-8 (Table 1) [27]. Similarly, Rv3033 is a secreted protein that is crucial for *Mtb* viability within macrophages [28]. Rv3033 has potent anti-apoptotic activity by suppressing the intrinsic apoptotic pathway. *M. smegmatis* expressing Rv3033 inhibits murine BMDM apoptosis compared with control strains. Rv3033 can actively block the translocation of pro-apoptotic Bax protein into mitochondria and cytochrome c into cytoplasm, thereby leading to suppress the caspase-9 activation (Table 1) [29]. Likewise, Rv3365c is another hypothetical protein that is an important *Mtb* anti-apoptotic effector. The deletion of Rv3365c significantly reduces the ability of *Mtb* to suppress U937 cells apoptosis in comparison with wild type strains. Rv3365c can revoke cellular apoptosis by interacting and inhibiting host cell membrane-bound serine protease cathepsin G and its downstream activation of caspase-1(Table 1) [30].

*Mtb* enhanced intracellular survival (Eis) protein is an important modulator of macrophage apoptosis, autophagy, and inflammatory cytokines production. In the context of apoptosis, Eis knockout *Mtb* strain significantly increases the murine BMDM when compared with wild type or complemented strains. Eis can repress JNK signaling leading to inhibition of ROS production (Table 1) [31].

*Mtb* stress response proteins play a significant role in bacterial resistance to the harsh milieu in both *in vitro* and in vivo [32,33]. *Mtb* SigH is a stress response regulon that controls the expression of multiple *Mtb* genes in response to oxidative and heat-stressors, as well as to host microenvironments [34,35]. Lower apoptosis of primary rhesus macaque bone marrow-derived macrophage (Rh-BMDM) was observed upon infection with SigH knockdown *Mtb* strains compared with wild *Mtb* strains [33]. SigH regulon can abolish cellular apoptosis through boosting prostaglandin synthetase 2 (PTGS2) expression, which in turn inhibits the P53-dependent apoptotic pathway (Table 1) [33].

*Mtb* Cpn60.2 (GroEL2) is a member of heat shock proteins found in the secreted form within the cytosol of macrophage [32]. Cpn60.2 can revoke THP-1 macrophage apoptosis through interacting with, and enhancing the stability of human mitochondrial Hsp70, which is (Table 1) an important anti-apoptotic factor [32] also known as mortalin [36].

*Mtb* acpM is a meromycolate extension acyl carrier protein involved in cell wall mycolic acid biosynthesis and a crucial drug target to eradicate multi-drugs-resistant *Mtb* strains (MDR) [37]. *M. smegmatis* expressing-acpM (Msm-acpM) can enhance the viability of BMDM in comparison with cells infected with *M. smegmatis,* harboring an empty vector. AcpM reduces ROS production by suppressing c-Jun N-terminal Kinase (JNK) signaling (Table 1) [38].

*Mtb* LpqT is a cell wall anchored lipoprotein that modulates macrophage responses by engaging with Toll-Like Receptor-2 (TLR-2) [39]. LpqT deleted *M. smegmatis* strains significantly induce higher levels of RAW264.7 macrophage apoptosis compared with control strains. LpqT could decrease the cleaved levels of caspase-3 by blocking the TLR-2 signaling pathway (Table 1) [39]. However, the previous study demonstrated that LpqT could increase the apoptosis of THP-1 macrophage and monocyte-derived macrophages (MDM) via agonist TLR-2 signaling [40]. Therefore, the exact role of *Mtb* LpqT in the regulation of macrophage apoptosis in the context of its interaction with TLR-2 remains controversial. It has been demonstrated that lipoprotein MPT83 can induce protective immunity against *Mtb* infection via promoting macrophage apoptosis [41]. 

EspR is an *Mtb* specific protein and a key regulatory protein of the ESX-1 secretion system and many other genes that are involved in *Mtb* pathogenesis [42]. Enforced expression of EspR in RAW264.7 macrophage leading to suppressed BCG mediating apoptosis. EspR can significantly decrease the cleavage of both caspase-3 and caspase-8 (Table 1) when compared with RAW264.7 harboring empty-vector. The mechanisms underlying caspase inhibition was due to EspR interfering with TLRs signaling by directly interacting with myeloid differentiated protein-88 (MyD88) [43].

## 3. Mycobacterium Thwarts Macrophage Apoptosis by Inducing Anti-Apoptotic Cytokines

**Figure 1:**
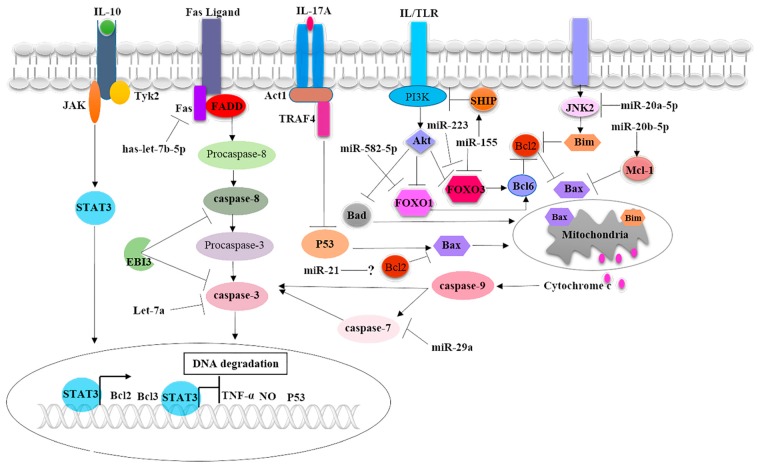
Mycobacterium evades macrophage apoptosis by induction of anti-apoptotic cytokines and miRNAs expression. Three cytokines, namely, IL-10, IL-17A, and EBI3, were demonstrated to play an anti-apoptotic role during *Mtb* infection. IL-10 can mediate the expression of anti-apoptotic Bcl2 and Bcl3 protein and suppresses the production of pro-apoptotic factors, including TNF-α, nitric oxide (NO), and P53. IL-17A signaling leads to suppression of P53 mediated Bax mitochondrial translocation. EBI3 can directly inhibit caspase-8 and caspase-3 activation. Nine anti-apoptotic miRs were found selectively regulated by *Mtb*. Has-let-7b-5p can target Fas leading to inhibition of downstream activation of caspase-3. Let-7a and miR-29a are targeting caspase-3 and caspase-7, respectively. MiR-155 can abolish expression of SH2 domain-containing inositol 5-phosphatase 1(SHIP1), leading to activation of phosphatidylinositol 3-kinase (PI3K) signaling-mediated inhibition of pro-apoptotic factors including Bad, FOXO-1 and 3. In addition, miR-155 can directly targeting and suppressing the expression of FOXO-3. MiR-582-5p can target the mRNA of FOXO-1 and block its expression. MiR-20a-5p can abrogate the activation of pro-apoptotic factor Bim by targeting the JNK-2 signaling pathway. However, miR-20b-5p can enhance the expression of anti-apoptotic factor Mcl-1, which can mediate block Bax mitochondrial translocation. The role of miR-21 in regulating anti-apoptotic Bcl-2 remains controversial.

Cytokines are inducible, soluble immune mediators responsible for orchestrating various and complex immunological processes, including immune cells crosstalk and apoptosis [44]. *Mtb* can differentially induce cytokines secretion to manipulate macrophage apoptosis. An earlier study showed that *Mycobacterium* could inhibit macrophage apoptosis by robust inducing interleukin-10 (IL-10) production, leading to the increased expression of Bcl2 and decreasing pro-apoptotic factors such as cleaved caspase-1, P53, nitric oxide (NO), and TNF-α production (Figure 1) [45]. Transgenic mice expressing human IL-10 in antigen-presenting cells (APCs) infected with *M. avium* displayed lower levels of macrophage apoptosis, which was accompanied by decreased TNF-α and NO production [46]. IL-10 also inhibits *Mtb* inducing alveolar macrophages (AM) apoptosis by abolishing TNF-α production. In this regard, IL-10 can increase the levels of B-cell lymphoma 3 protein (Bcl-3), which in turn antagonizes TNF-α production via deactivating NF-kappaB nuclear signaling [47].

IL-17A, a cytokine secreted in high amount by T helper-17 (Th17) in response to *Mtb* infection and actively contributes to TB pathogenesis [48]. It has been shown that the exogenous addition of IL-17A can significantly enhance BCG strains and *Mtb* viability within murine BMDM when compared with bacterial intracellular growth in the absence of IL-17A. This cytokine can blockade BMDM apoptosis upon BCG infection by suppression of intrinsic apoptotic pathway through interfering with nuclear translocation of P53, thereby leading to the upregulation of Bcl2 expression and downregulation of Bax expression, as well as abolishing cytochrome c released and caspase-3 activation (Figure 1) [49].

Epstein–Barr virus-induced gene 3 (EBI3), is a subunit of anti-inflammatory cytokines, namely IL-27 and IL-35. It was found that EBI3 is produced in a significant amount in CD14+ macrophages isolated from TB patients compared to cells from healthy donors. Furthermore, it has been demonstrated that EBI3 production and accumulation were markedly increased upon the challenge of murine peritoneal macrophages with *Mtb*, suggesting that it may contribute to the immunoevasion of macrophage responses. *Mtb* and BCG strains shown to induce significant apoptosis of murine EBI3 deleted macrophages in comparison with the deaths of wild type cells. EB13 abrogates the extrinsic apoptotic pathway via suppression of the cleavage of caspase-3 and caspase-8 (Figure 1) [50]. Taken together, *Mtb* can stimulate IL-10, IL-17A, and EBI3 secretion to frustrate macrophage apoptosis. Thus, complementary to antibodies blocking the action of these cytokines, anti-tuberculous drugs can hasten the eradication of bacilli and shortage of treatment regimens.

## 4. *Mtb* can Suppress Apoptosis by Regulating microRNAs Expression

MicroRNAs (miRs) are small noncoding RNAs that play an indispensable role in posttranscriptional regulation of genes expression that involved in various cellular biochemical pathways, including immune signaling pathways in response to pathogenic infections such as *Mtb* [51].

*Mtb* can selectively regulate miRs expression to defeat macrophages apoptosis in order to enhance its intracellular viability and growth. Consistently, miR-582-5p has been significantly more upregulated in monocytes obtained from active TB patients than the cells from healthy controls. The transfection of THP-1 monocytic cells with miR-582-5p mimics leads to the blocking of apoptosis when compared cells transfected with the negative control. MiR-582-5p can inhibit monocytes apoptosis by directly targeting forkhead box O1 (FOXO1) mRNA and suppressing its translation (Figure 1) [52].

MiR-155 expression significantly upregulated in the peripheral blood monocytes (PBMCs) obtained from patients with active TB disease than the cells from healthy control [53]. The apoptosis of CD4+ monocytes was significantly decreased in active TB patients compared with healthy control, suggesting that miR-155 may abolish cellular apoptosis. The ectopic expression of miR-155 in THP-1 macrophages leading to abrogate BCG-inducing apoptosis via downregulating FOXO3 expression (Figure 1) [53]. Furthermore, *Mtb* infected miR-155 knockdown murine macrophages exhibited a higher level of apoptosis when compared with wild type macrophages infected with *Mtb* [54]. MiR-155 can target the mRNA of SH2 domain-containing inositol 5-phosphatase 1(SHIP1), which was confirmed by increased protein levels in miR-155 deleted macrophages. Increased levels of SHIP1 can result in reduced phosphorylation of Akt, thereby leading to the enhanced activation of Bad, FOXO-1, 3, and caspase-3 (Figure 1) [54].

MiR-223 is expressed significantly more in peripheral macrophages harvested from active TB patients compared with cells from healthy controls and upregulated during the in vitro *Mtb* infection of human macrophages. The forced expression miR-223 in human macrophages significantly reduced the rate of apoptosis in comparison with macrophages transfected with control miR-223 mimics. Mechanistically, miR-223 blocks macrophage apoptosis by targeting FOXO3 and markedly reducing its protein expression (Figure 1) [55]. 

Let-7e and miR-29a are highly expressed in human monocyte-derived macrophages in response to *M. avium* infection. They block macrophages apoptosis by repressing the expression of caspase-3 and 7, which are direct targets for let-7e and miR-29a, respectively (Figure 1) [56].

MiR-21 expression was upregulated by NF-κB signaling in RAW264.7 murine macrophage triggered by *Mtb* mpt68 protein. It has been demonstrated that miR-21 could inhibit murine macrophage apoptosis by positively regulating anti-apoptotic Bcl-2 expression (Figure 1) [57]. However, recent studies have demonstrated that miR-21 enhances macrophages apoptosis in response to mycobacterium infection by negatively regulating the expression of Bcl-2 [58,59]. Therefore, the role of miR-21 in regulating macrophage apoptosis and Bcl-2 expression remains ambiguous.

MiR-20a-5p expression was found to be downregulated in CD14+ monocytes from active pulmonary TB patients, while it is pronouncedly upregulated by successful anti-TB treatment. The downregulation of miR-20a-5p expression also observed during the *Mtb* infection of THP-1 human macrophages. Blockade of miR-20a-5p expression can accelerate THP-1 macrophage apoptosis and promote mycobacterium survival. MiR-20a-5p ectopically expressed in THP-1 cells led to the decreased expression of pro-apoptotic gene Bim through targeting and abolishing the expression of JNK2 signaling (Figure 1) [60]. Recently, the downregulation of miR-20b-5p expression has been reported upon the *Mtb* infection of RAW264.7 macrophages. The overexpression of miR-20b-5p can promote bacilli survival and cell apoptosis [61]. In this regard, miR-20b-5p can increase macrophage apoptosis by targeting and suppressing the expression of the anti-apoptotic Mcl-1 gene (Figure 1) [61], which is a member of the Bcl-2 protein family.

Hsa-let-7b-5p was significantly upregulated in *Mtb* infected THP-1 macrophages, leading to the subversion of apoptosis. Mechanistically, hsa-let-7b-5p negatively regulates the expression of Fas by directly targeting its mRNA, which can lead to blocking pathways of caspase-3 activation (Figure 1) [62].

## 5. Conclusions

Macrophages are the first line of innate defense against *Mtb* infection, and the subversion of macrophage apoptosis is considered a hallmark of *Mtb* pathogenesis. *Mtb* is a mystery pathogen that utilizes multiple strategies to abolish apoptotic signaling pathways in order to establish persistent infection. The impairment of macrophage apoptosis leads to the enhanced intracellular survival of bacilli and compromises the cell-mediated immune response. *Mtb* has a broad spectrum of anti-apoptotic effector molecules that direct targeting cellular pro-apoptotic factors or block the signaling that regulates their expression. Some *Mtb* anti-apoptotic effectors, including PE_PGRS18 and IcI, impaired macrophage apoptosis by unrecognized mechanisms. Moreover, the role of *Mycobacterium* lipoprotein (LpqT) in modulating macrophage apoptosis needed to be clarified. Future studies are needed to identify unrevealed mechanisms of *Mtb* anti-apoptotic effectors and to develop methods able to hamper *Mtb* effectors’ capability to abrogate macrophage apoptosis.

*Mtb* can also manipulate macrophage apoptosis by selectively regulating cytokines and miRs expression. It is also important to understand how *Mtb* selectively regulates anti-apoptotic cytokines and miRs expression. The mechanisms by which cytokines and miRs enhance the viability of macrophages are dependent on the aberrant activity of key pro-apoptotic regulators, including P53, TNF-α, Fas, FOXO, and Bad. They promote the activity of anti-apoptotic regulators such as SHIP, Bcl-2, and Mcl-1. Taken together, these insights into the intelligent subversion mechanisms of macrophage apoptosis by *Mtb* elucidate promising and novel therapeutic targets to eliminate the intracellular survival of bacilli and promote an adaptive immune response.

## Figures and Tables

**Table 1:** Anti-apoptotic effectors of *Mycobacterium.*

Effector	Cell Model	Mechanisms	Outcome	References
ManLAM	B10R	Blocks Ca^+2^ influx to the cells.	Inhibition of caspase-1 cleavage, alter mitochondrial membrane permeability and upregulate Bcl-2	Rojas et al., 2000
LAM	THP-1	Activation of PI3K signaling	Suppression of Bad	Maiti et al., 2001
PtpA	THP-1	Dephosphorylation of GSK3	Inhibition of caspase-3 cleavage	Poirier et al., 2014
	U937	Suppress ubiquitin ligase activity of the TRIM protein	Inhibition of caspase-3 cleavage	Wang et al., 2016
MptpA	RAW264.7	Reduction of P53 levels	Inhibition of caspase-3 cleavage	Fan et al., 2018
PknE	THP-1	Phosphorylation of Akt	Inhibition of Bad	Kumar and Narayanan et al., 2012
		Inhibit the expression of pro-apoptotic factors, including P53, TNF-α and Bax	Inhibition of caspase-3 activation	
		Promote the anti-apoptotic factor Mcl-1 expression	Block Bax mitochondrial translocation	
NuoG	THP-1 and BMDM	Blocks of NADPH oxidase mediating ROS production	Inhibition of TNF-α production	Miller et al., 2010
Ndk	RAW264.7	Inhibit NOX2 assembly and ROS production	Inhibition of caspase-3 activation	Sun et al., 2013
Icl	RAW264.7	Unknown	Unknown	Li et al., 2008
PE_PGRS62	THP-1	Suppression of pro-apoptotic stress-response genes expressions such as CHOP and GRP78/Bip	Inhibit endoplasmic reticulum (ER) stress response	Long et al., 2019
PE_PGRS41	THP-1	Uncertain	Reduction the cleavage level of caspase 3 and 9	Deng et al., 2017
PE_PGRS18	THP-1	Unknown	Unknown	Yang et al., 2017
Rv3654c Rv3655c	U937	Degrade the polypyrimidine tract binding PSF	Suppression of caspase-8 activation	Danelishvili et al., 2010
Rv3033	RAW264.7 and murine BMDM	Abolish translocation of Bax into mitochondria and cytochrome c into cytoplasm	Suppression of caspase-9 activation	Zhang et al., 2018
Rv3365c	U937	Inhibit serine cathepsin G	Suppression of caspase-1	Danelishvili et al., 2012
Eis	Murine BMDM	Block the JNK signaling	Inhibition of ROS production	Shin et al., 2010
SigH	Rh-BMDM	Promote prostaglandin synthetase-2 expression	Inhibition P53 dependent pathway	Dutta et al., 2012
AcpM	Murine BMDM	Suppress JNK signaling	Reduction of ROS production	Paik et al., 2019
LpqT	RAW264.7	Antagonized TLR-2 signaling	Inhibition of caspase-3 cleavage	Li et al., 2018
EspR	RAW264.7	Block TLR signaling	Inhibition of caspase-8 and 3 cleavage	Jin et al., 2019

PI3K, phosphatidylinositol 3-kinase; GSK3, glycogen synthetase-3; TRIM, tripartite motif; CHOP, C/EBP homologous protein; GRP78/Bip, 78-kDa glucose-regulated protein; PSF, Protein-associated Splicing Factor; TLR, Toll-Like Receptor; JNK, c-Jun N-terminal Kinase; BMDM, bone marrow-derived macrophages; Rh, Rhesus.
